# Assessing the quality of health research from an Indigenous perspective: the Aboriginal and Torres Strait Islander quality appraisal tool

**DOI:** 10.1186/s12874-020-00959-3

**Published:** 2020-04-10

**Authors:** Stephen Harfield, Odette Pearson, Kim Morey, Elaine Kite, Karla Canuto, Karen Glover, Judith Streak Gomersall, Drew Carter, Carol Davy, Edoardo Aromataris, Annette Braunack-Mayer

**Affiliations:** 1 Wardliparingga Aboriginal Health Research Unit, South Australian Health and Medical Research Institute, PO Box 11060, Adelaide, South Australia 5001 Australia; 2 School of Public Health, The University of Adelaide, Adelaide, Australia; 3 Healthy Mothers, Babies and Children, South Australian Health and Medical Research Institute, Adelaide, Australia; 4 Murdoch Children’s Research Institute, Melbourne, Australia; 5 Joanna Brigg Institute, The University of Adelaide, Adelaide, Australia; 6 Adelaide Health Technology Assessment, The University of Adelaide, Adelaide, Australia; 7 School of Health and Society, University of Wollongong, Wollongong, Australia

**Keywords:** Aboriginal and Torres Strait Islander people, Indigenous, Australia, Indigenous epistemologies, Quality appraisal, Systematic reviews, Meta-syntheses

## Abstract

**Background:**

The lack of attention to Indigenous epistemologies and, more broadly, Indigenous values in primary research, is mirrored in the standardised critical appraisal tools used to guide evidence-based practice and systematic reviews and meta-syntheses. These critical appraisal tools offer no guidance on how validity or contextual relevance should be assessed for Indigenous populations and cultural contexts. Failure to tailor the research questions, design, analysis, dissemination and knowledge translation to capture understandings that are specific to Indigenous peoples results in research of limited acceptability and benefit and potentially harms Indigenous peoples. A specific Aboriginal and Torres Strait Islander Quality Appraisal Tool is needed to address this gap.

**Method:**

The Aboriginal and Torres Strait Islander Quality Appraisal Tool (QAT) was developed using a modified Nominal Group and Delphi Techniques and the tool’s validity, reliability, and feasibility were assessed over three stages of independent piloting. National and international research guidelines were used as points of reference. Piloting of the Aboriginal and Torres Strait Islander QAT with Aboriginal and Torres Strait Islander and non-Indigenous experts led to refinement of the tool.

**Results:**

The Aboriginal and Torres Strait Islander QAT consists of 14 questions that assess the quality of health research from an Aboriginal and Torres Strait Islander perspective. The questions encompass setting appropriate research questions; community engagement and consultation; research leadership and governance; community protocols; intellectual and cultural property rights; the collection and management of research material; Indigenous research paradigms; a strength-based approach to research; the translation of findings into policy and practice; benefits to participants and communities involved; and capacity strengthening and two-way learning. Outcomes from the assessment of the tool’s validity, reliability, and feasibility were overall positive.

**Conclusion:**

This is the first tool to appraise research quality from the perspective of Indigenous peoples. Through the uptake of the Aboriginal and Torres Strait Islander QAT we hope to improve the quality and transparency of research with Aboriginal and Torres Strait Islander peoples, with the potential for greater improvements in Aboriginal and Torres Strait Islander health and wellbeing.

## Background

There are approximately 370 million Indigenous peoples, living in 90 countries and comprising 5 % of the world’s population [1]. Diverse in culture, practices, language, knowledges, and beliefs, they are the world’s longest surviving peoples. However, many are now the most marginalised communities in the world, living in poverty with minimal or no access to education and social and health services [2]. Indigenous peoples are also, per capita, among the most researched communities in the world [3, 4], with the bulk of this research conducted by non-Indigenous researchers. Increasingly, Indigenous community leaders and organisations have called for research ‘on’ Indigenous peoples to end, challenging researchers to acknowledge that significant benefits for Indigenous peoples will only come with meaningful partnerships between researchers and Indigenous peoples [5].

Recent national [6–9] and international [10] research guidelines reflect these calls for more equal, collaborative and culturally sensitive partnerships between researchers and Indigenous peoples. They emphasise the need for researchers to work with Indigenous communities to identify appropriate research questions, to design and conduct studies, to disseminate findings, and to translate findings into practice [11, 12]. Despite this progress and the existence of some outstanding research practices, much research in the health and social sciences still fails to partner with Indigenous peoples and organisations and thereby fails to meet Indigenous people’s real needs. This is particularly evident in health research where non-Indigenous researchers continue to work in Indigenous settings with relatively little input from the people they are researching [13–15].

Meaningful partnerships are hard to achieve when Indigenous communities and non-Indigenous researchers differ on what constitutes knowledge, how it is acquired and how it is used. These epistemological issues are profoundly important [16–21]. For example, in Australia, health research with Aboriginal and Torres Strait Islander peoples continues to ignore the importance of their relationships with each other and with country [11]. Meanwhile, in Canada, Indigenous organisations have challenged deficit-based research, which focusses on documenting problems, and demanded instead that research be strengths-based [22].

To conduct research that is respectful and credible, researchers need to privilege Indigenous epistemologies [17, 18]. Failure to tailor the research questions, design, analysis, dissemination and knowledge translation towards capturing understandings that are specific to Indigenous peoples results in research of limited acceptability and benefit and potentially harms Indigenous peoples [23–26].

The lack of attention to Indigenous epistemologies and, more broadly, Indigenous values and principles in primary research is mirrored in the standardised critical appraisal tools used to guide evidence-based practice and systematic reviews and meta-syntheses. These tools offer no guidance on how validity or contextual relevance should be assessed for different Indigenous populations and cultural contexts. Specifically, existing critical appraisal tools fail to reflect Aboriginal and Torres Strait Islander values and principles for ethical research, such as reciprocity, responsibility, survival and protection, equality, and respect for the communities involved in the research [11, 27].

We sought to develop and trial a tool to assess the quality of research from an Indigenous perspective, specifically health research involving Aboriginal and Torres Strait Islander peoples in Australian settings.

## Methods

In November 2013, the newly established Australian National Health and Medical Research Council (NHMRC) funded Centre of Research Excellence in Aboriginal Chronic Disease Knowledge Translation and Exchange (CREATE) formed a Methods Group to enhance existing systematic review method guidance from an Aboriginal and Torres Strait Islander perspective. The CREATE Methods Group consisted of 11 researchers with expertise in public health, ethics, biomedical and clinical research, and systematic reviews – six senior Aboriginal and Torres Strait Islander researchers (SH, OP, KM, EK, KC, KG) and five non-Indigenous researchers (JGS, DC, CD, EA, ABM).

**Fig. 1:**
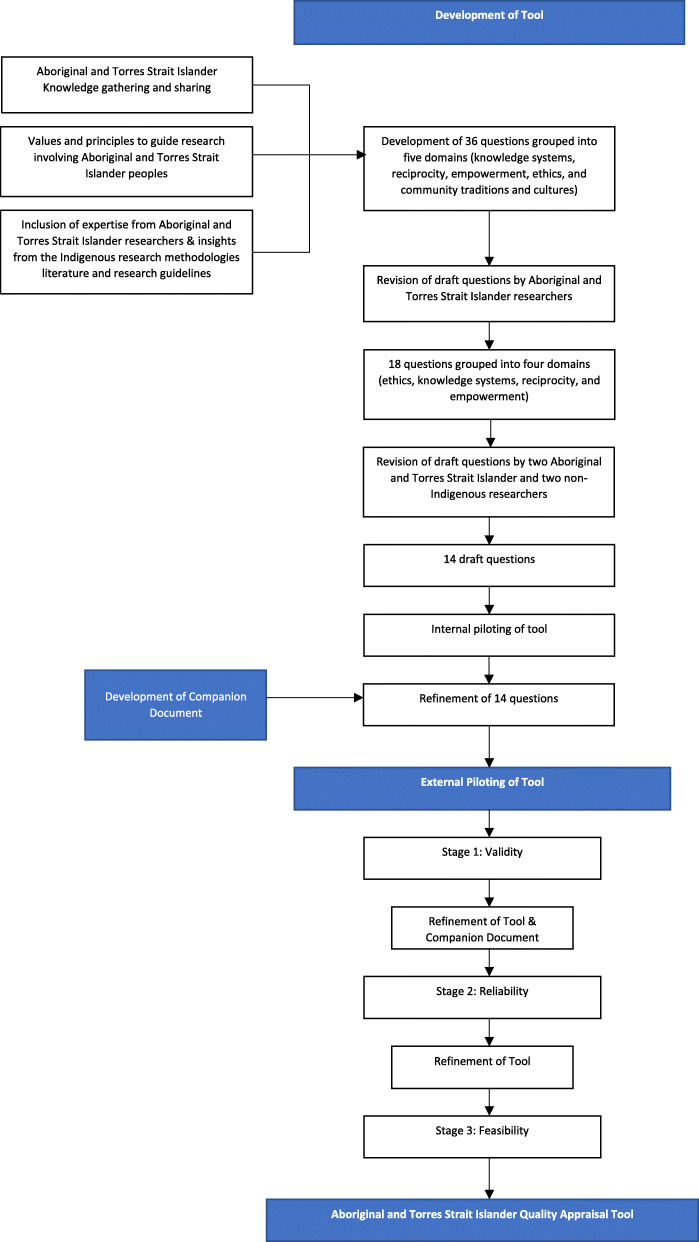
Process of the Development of the Aboriginal and Torres Strait Islander QAT

Initially, three group meetings were held to discuss the nature and purpose of knowledge gathering and sharing amongst Aboriginal and Torres Strait Islander peoples; and, the role of systematic reviews and critical appraisal in Aboriginal and Torres Strait Islander research (Fig. 1). We agreed on a single question to guide our work:

Over 12 months, we met 10 times, supported by regular email contact in between, to draft a set of core values and principles to guide research involving Aboriginal and Torres Strait Islander peoples. Our intention was to develop a tool that would complement and not replicate other critical appraisal tools. We began by using a modified nominal group technique [28, 29] to canvas immediate responses to our guiding question. We incorporated the expertise of the Aboriginal and Torres Strait Islander researchers, insights from the Indigenous research methodologies literature [19, 20] and key Australian Aboriginal and Torres Strait Islander research guidelines, including the NHMRC’s *Values and Ethics: Guidelines for Ethical Conduct in Aboriginal and Torres Strait Islander Health Research* [8] and the *South Australian Aboriginal Health Research Accord* [27]. By the end of this process, we had identified, described and agreed on 36 items phrased as questions and grouped into five domains (knowledge systems, reciprocity, empowerment, ethics, and community traditions and cultures).

What are the principal features of ethical Indigenous methodologies?

The next stage saw the Aboriginal and Torres Strait Islander researchers review the 36 draft questions for their importance and uniqueness over six meetings. This process involved reformulating questions for clarity and consolidating similar questions. Eighteen questions were retained under four domains (ethics, knowledge systems, reciprocity, and empowerment). The questions agreed on by the Aboriginal and Torres Strait Islander researchers were presented back to the non-Indigenous researchers and together were reviewed for interoperability. During this process, the questions were again reviewed against two key Australian Aboriginal and Torres Strait Islander research guidelines [8, 27]. Finally, two Aboriginal and Torres Strait Islander researchers and two non-Indigenous researchers reviewed the questions again to ensure that they were unique, easy to interpret, and could be answered with a simple ‘yes’, ‘partially’, ‘no’, or ‘unsure’. The whole group then refined the tool yet again and reduced it to 14 questions. We piloted the tool using three articles [30–32] and made minor adjustments. During these stages, we also developed a Companion Document to provide guidance on understanding and answering the questions (Supplementary file 1). The Companion Document was included in subsequent piloting of the tool.

### Piloting the Aboriginal and Torres Strait Islander QAT

We used a modified Delphi technique [28, 29] to assess the tool’s content validity, test-retest reliability, and feasibility. We invited external independent Aboriginal and Torres Strait Islander and non-Indigenous researchers with experience in conducting Aboriginal and Torres Strait Islander health research and or conducting systematic reviews to be involved in the three stages of independent piloting.

In the first stage, we selected Aboriginal and Torres Strait Islander researchers with experience in conducting Aboriginal and Torres Strait Islander health research to assess the tool for content validity. They critiqued the tool for its meaningfulness and comprehensiveness, attending to its language, organisation and the uniqueness of the 14 questions (Supplementary file 2).

The second stage of independent piloting involved another selected group of Aboriginal and Torres Strait Islander and non-Indigenous researchers with experience in conducting systematic reviews. They appraised two articles using the tool (Supplementary file 3). Test-retest reliability using per cent agreement was assessed with the same participants reviewing the same two articles 2 weeks later.

Finally, in stage three, the tool’s feasibility was assessed by a third group of Aboriginal and Torres Strait Islander and non-Indigenous researchers with experience in conducting systematic reviews. Each researcher used the tool in conjunction with another critical appraisal tool of their choice to appraise two out of six articles sent to them (Supplementary file 3). They also completed a questionnaire on the feasibility of using the tool on its own (Supplementary file 4) and in conjunction with another critical appraisal tool of their choice.

Ethics approval was given by the Aboriginal Human Research Ethics Committee (Aboriginal Health Council of South Australia) [Ref. No: 04–16-666].

## Results

### The Aboriginal and Torres Strait Islander Quality Appraisal Tool

**Fig. 2:**
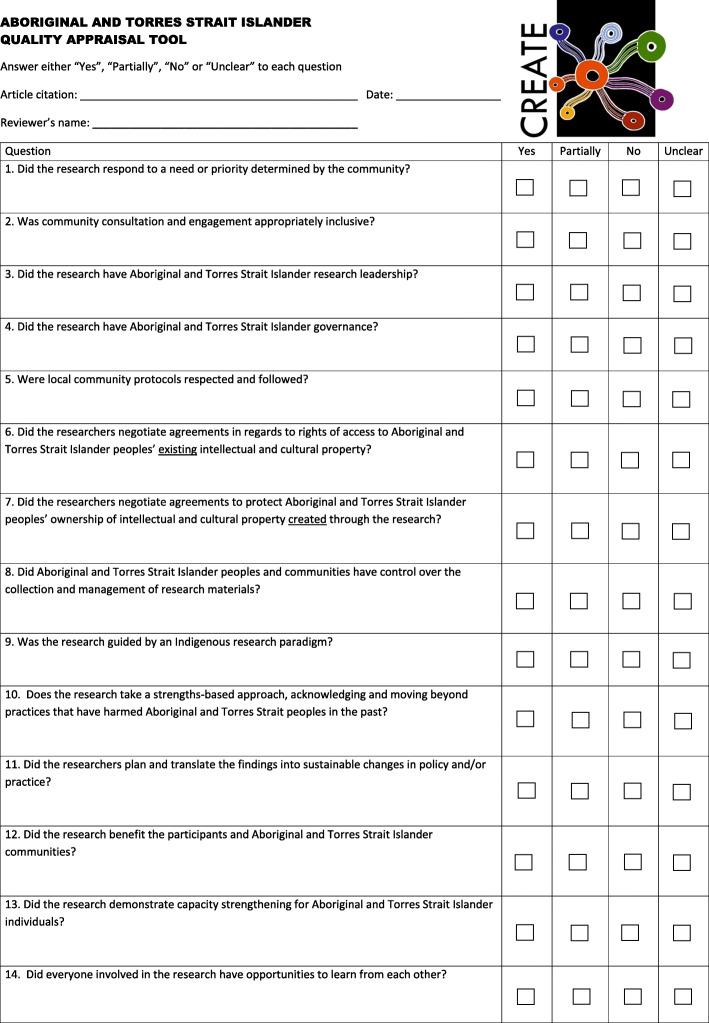
The Aboriginal and Torres Strait Islander Quality Appraisal Tool

The Aboriginal and Torres Strait Islander Quality Appraisal Tool (the Aboriginal and Torres Strait Islander QAT) consists of 14 questions that assess the quality of health research from an Aboriginal and Torres Strait Islander perspective (Fig. 2). The questions encompass matters such as setting appropriate research questions; community engagement and consultation; research leadership and governance; community protocols; intellectual and cultural property rights; the collection and management of research material; Indigenous research paradigms; a strength-based approach to research; the translation of findings into policy and practice; benefits to participants and communities involved; and capacity strengthening and two-way learning. Each question is to be answered with a ‘Yes’, ‘Partially’, ‘No’, or ‘Unclear’, with space provided for the user to record comments about their decision. We also developed a Companion Document, which provides guidance on understanding and applying the Aboriginal and Torres Strait Islander QAT (Supplementary file 1).

### Pilot experience with the Aboriginal and Torres Strait Islander QAT

Six independent researchers, all of whom were Aboriginal and/or Torres Strait Islander, participated in stage-one piloting, which focussed on content validity. Participants found the Aboriginal and Torres Strait Islander QAT comprehensive, the language and organisation appropriate, and the questions unique. They also supported weighting the importance of the questions equally. In addition, the participants proposed several amendments, including changing ‘Unsure’ to ‘Unclear’ to account for studies for which there was insufficient detail, and placing greater emphasis on the holistic nature of Aboriginal and Torres Strait Islander views of health and research. In this stage, participants also provided feedback on the Companion Document. They suggested providing definitions for terms such as ‘community’, ‘cultural and intellectual property’, and ‘Indigenous research paradigm’. They also suggested enhancing the Companion Document’s preamble to include statements on strength-based approaches to research, on complex issues that often require multiple and unique strategies, and on how the Aboriginal and Torres Strait Islander QAT was developed and should be used. All suggestions for refinement of the Aboriginal and Torres Strait Islander QAT were considered and included.

A further six independent researchers, two of whom were Aboriginal, participated in stage two, which focused on test re-test reliability. For paper one, in total a 73% agreement was reached among the six independent researchers, individually per cent agreement ranged from 50 to 100%. For paper two, in total a 67% agreement was reached, individually precent agreement ranged from 36 to 93%. Among the two papers, individual questions which received a per cent agreement of less than 67% were questions 9, 10, 12, 13 and 14. Overall, this indicates that when reassessing articles using the Aboriginal and Torres Strait Islander QAT, there was some variation in how participants answered questions. However, based on these results no questions were excluded from the Aboriginal and Torres Strait Islander QAT. Further explanation of these results is discussed below.

Finally, seven independent researchers, two of whom were Aboriginal, participated in stage three, which focused on feasibility. Participants took 15–50 min to read and appraise each article, needing a similar length of time to read and assess each article using a critical appraisal tool of their choice (Supplementary file 5). Participants reportedly found it easy to assess each article using the Aboriginal and Torres Strait Islander QAT, particularly with the support of the Companion Document. Participants reported that using both appraisal tools together was sensible and complementary, with there being no overlap between them. One participant commented as follows:

*Used alongside a standard Western research quality perspective the tool substantially improves the critical appraisal of papers reporting on Aboriginal and Torres Strait Islander health by turning reviewers' minds to those matters that need to be considered but are missing from the standard tools. [Participant 03-01]*

Additional suggestions related to providing a space for assessor comments for each question.

## Discussion

The Aboriginal and Torres Strait Islander QAT consists of 14 questions that assess the quality of research from an Aboriginal and Torres Strait Islander perspective, and it should be used in conjunction with other appropriate appraisal tools. Guidance for using the Aboriginal and Torres Strait Islander QAT is provided in the Companion Document (Supplementary file 1). At a minimum, the results from appraisal should include a summary of each question with the number of papers that were assessed as either yes, partially, no or unclear. Reviewers may choose to highlight particular questions that rated poorly or well.

Until now, quality appraisal tools have used western research principles and ideals to assess the rigour of the study design and the appropriateness of methods. These tools do not have the scope to appraise studies that have used an Indigenous methodology. For example, they do not assess whether Indigenous participants maintained control over their cultural knowledge or whether the research was guided by an Indigenous governance structure [33].

To our knowledge, this is the first tool to appraise research quality from the perspective of Indigenous peoples. We identified only one other tool, by Ritte et al. [34, 35], a data extraction and quality assessment/risk of bias tool that includes elements about Aboriginal and Torres Strait Islander peoples’ involvement in the research. Commendably, that tool was designed by a team of Indigenous and non-Indigenous researchers who drew on existing systematic review tools as well as the NHMRC’s *Values and Ethics: Guidelines for Ethical Conduct in Aboriginal and Torres Strait Islander Health Research* [8]. The Ritte tool was trialled by the team who developed it but not externally. The Aboriginal and Torres Strait Islander QAT is more thorough and comprehensive in its assessment of research quality from an Indigenous perspective. In addition, the tool is applicable to the full breadth of research conducted with Aboriginal and Torres Strait Islander peoples and can be used in conjunction with existing standardised critical appraisal tools.

The development of the Aboriginal and Torres Strait Islander QAT had several strengths. First, it embodied a research paradigm that reflects Aboriginal and Torres Strait Islander ways of knowing, being and doing and is based on the lived experiences and knowledges of Aboriginal and Torres Strait Islander peoples. It therefore reflects the values, priorities and perspectives of Aboriginal and Torres Strait Islander peoples and their communities. Second, the tool was developed through partnership between Aboriginal and Torres Strait Islander and non-Indigenous team of researchers, and it privileged the Indigenous epistemologies of the Aboriginal and Torres Strait Islander researchers of the team. Third, the Aboriginal and Torres Strait Islander QAT was explicitly informed by existing national ethical guidelines [8, 27]. Finally, the Aboriginal and Torres Strait Islander QAT underwent rigorous piloting with both Aboriginal and Torres Strait Islander and non-Indigenous researchers to assess validity, reliability and feasibility.

Although the outcomes of the pilot were positive, results for test-retest reliability weren’t perfect. We decided not to exclude questions from the Aboriginal and Torres Strait Islander QAT based on the test-retest reliability results. Individual questions which received a per cent agreement of less than 67% included questions that constitute important concepts of Indigenous research methodology – Indigenous research paradigm, strengthen based approach, benefit, capacity strengthening and two-way learning; and are essential for assessing research quality in this context, as indicated by results from stage one – content validity. Furthermore, this is the first appraisal tool of its kind and, for most participants, it is possibly the first time that they have ever explicitly assessed literature from an Indigenous perspective. In addition, many of the questions in the Aboriginal and Torres Strait Islander QAT are not questions that reviewers would typically ask or that researchers would typically think to report. Third, while we have provided thorough guidance on how to understand the questions in the Aboriginal and Torres Strait Islander QAT, questions such as ‘was the research guided by an Indigenous research paradigm?’ feature concepts that are difficult to grasp. Finally, the notion of reliability itself is a product of the Western positivist tradition. Indigenous epistemologies do not necessarily assume that getting the same answer when something is repeated is good, or even relevant. Additionally, familiarity and ease of use of the Aboriginal and Torres Strait Islander QAT will improve as the tool is used by researchers.

We expect that use of the Aboriginal and Torres Strait Islander QAT will improve systematic reviews, meta-syntheses and evidence-based practice in Australia by allowing studies to be appraised for additional research qualities and values important to Aboriginal and Torres Strait Islander peoples. The tool can also be used by editors to judge whether research should be published; to guide funding requirements and applications; to inform the development of research reporting guidelines; and to educate researchers about how to conduct respectful, high-quality research with Aboriginal and Torres Strait peoples and communities. Finally, and most importantly, the Aboriginal and Torres Strait Islander QAT, in giving voice to Aboriginal and Torres Strait Islander ways of understanding research, can contribute to purposes that extend beyond improving the quality and outcomes of research practice; it can also “decolonize, rebalance power, and provide healing” [21].

It is critical that research with Aboriginal and Torres Strait Islander peoples continues to improve and reflect the values, priorities and perspectives of the Aboriginal and Torres Strait Islander peoples and communities involved in the research. To provide the most benefit, research must be conducted respectfully and appropriately, occur in equal partnership with Aboriginal and Torres Strait Islander peoples and communities, and result in meaningful findings that are translated into policy and practice.

Further refinement of the Aboriginal and Torres Strait Islander QAT and Companion Document will continue, and we invite feedback. In addition, we invite conversations with other Indigenous peoples interested in developing a tool for Indigenous peoples globally.

### Limitations

The Aboriginal and Torres Strait Islander QAT was specifically developed to apply to studies about Aboriginal and Torres Strait Islander peoples. The Aboriginal and Torres Strait Islander QAT may not be suitable for other studies conducted with or including other Indigenous populations. The Aboriginal and Torres Strait Islander QAT is not a stand-alone tool and can be used with other suitable critical appraisal tools that focus on rigour of the study design and the appropriateness of methods that draw on western research principles. This is also a strength of the Aboriginal and Torres Strait Islander QAT, since it does not replicate other critical appraisal tools. Although we have offered explanations for the results of the test-retest reliability testing, it remains inconsistent. Additionally, while content validity was assessed, participants were not asked to provide an individual assessment of the necessity of each question, so a content validity index is not able to be calculated. However, individuals were asked to comment on the overall completeness of the tool in assessing ethical and methodological issues relating to research involving Aboriginal and Torres Strait Islander people. As use of the Aboriginal and Torres Strait Islander QAT becomes more practised and widespread, reliability may improve.

## Conclusion

The Aboriginal and Torres Strait Islander QAT has been developed to assess the quality of health research from an Aboriginal and Torres Strait Islander perspective. It offers a tool that privileges Indigenous epistemologies, values and principles for ethical research. Through the uptake of the Aboriginal and Torres Strait Islander QAT we hope to improve the quality and transparency of research with Aboriginal and Torres Strait Islander peoples, with the potential for greater improvements in Aboriginal and Torres Strait Islander health and wellbeing.

## Supplementary information

**Additional file 1.**

**Additional file 2.**

**Additional file 3.**

**Additional file 4.**

**Additional file 5.**

## Data Availability

All data generated or analysed during this study is not publicly available as it is confidential and cannot be shared.

## Abbreviations

CREATE: Centre of Research Excellence in Aboriginal Chronic Disease Knowledge Translation and Exchange
NHMRC: National Health and Medical Research Council
QAT: Quality appraisal tool
