# Supplementary material for: Assessing the quality of health research from an Indigenous perspective: the Aboriginal and Torres Strait Islander quality appraisal tool
Source: BMC Med Res Methodol. 2020 Apr 10;20:79. doi: 10.1186/s12874-020-00959-3 (PMC7147059; doi:10.1186/s12874-020-00959-3)
Supplement: Supplementary file 2 — Additional file 2. [file 12874_2020_959_MOESM2_ESM.pdf]

QUESTIONNAIRE FOR STAGE 1 OF THE PILOTING A TOOL TO CRITICALLY APPRAISE LITERATURE THROUGH AN ABORIGINAL AND TORRES STRAIT ISLANDER LENS STUDY

Thank you for participating in the validation of the CREATE Critical Appraisal Tool and user guide. This Appraisal Tool is designed to be used in conjunction with relevant standardised tools for appraising research (i.e. that appraise from a Western perspective of research quality). Hence the criteria in this Appraisal Tool are designed to capture methodological and ethical issues that are unique to credible and ethical research involving Aboriginal and Torres Strait Islander Australian participants.

Your expert knowledge will be critical in helping to ensure that the appraisal tool includes criteria that comprehensively and appropriately assess quality of research involving Aboriginal and Torres Strait Islander peoples, from an Aboriginal and Torres Strait Islander perspective. Please read the DRAFT CREATE CRITICAL APPRAISAL TOOL and user guide, then answer the following questions. When you have completed the questions, please send the completed questionnaire to Stephen Harfield, CREATE Research Fellow on email: stephen.harfield@sahmri.com.

Participant ID:

Comprehensiveness of the criteria

1. Do the questions in the tool comprehensively address all the unique ethical and methodological issues relating to research involving Aboriginal and Torres Strait Islander participants in Australia?

   If you answered no to question 1, please recommend additional questions that should be added to ensure that the tool adequately assesses research quality from an Aboriginal and Torres Strait Islander perspective, with an explanation as to why you believe these changes are necessary.

Uniqueness of each criteria

2. Is there any duplication in the tool, in that more than one question addresses the same issue?

   If you answered yes to question 2, please explain what question(s) you would remove from the tool and why.

Language and organisation of the questions

3. Do any of the questions need further clarification?

   If you answered yes to question 3, please explain which question(s) and how you would clarify the questions?

4. Does the organisation of the questions need to be altered (please explain motivation for any recommended changes)?
   If you answered yes to question 4, please explain how you would organise the question(s) and why.

Assessment approach

5. Is it appropriate for all the questions/criteria on the tool to be assessed by users as "Yes" "No" or "Partially"?
   If you have answered no to question 5, please explain which questions require a different approach, suggest what the alternative approach should by and why.

6. Should each of the questions be given equal weight?
   If you answered no to question 6, please propose an alternative weighting system (rather than all questions equal weight) and motivate for it.
