# Supplementary material for: Assessing the quality of health research from an Indigenous perspective: the Aboriginal and Torres Strait Islander quality appraisal tool
Source: BMC Med Res Methodol. 2020 Apr 10;20:79. doi: 10.1186/s12874-020-00959-3 (PMC7147059; doi:10.1186/s12874-020-00959-3)
Supplement: Supplementary file 3 — Additional file 3. [file 12874_2020_959_MOESM3_ESM.pdf]

Supplementary File 3 – Articles Used in Stages 2 and 3 of Piloting

**Stage 2**
Hinton R, Kavanagh DJ, Barclay L, Chenhall R, Nagel T. Developing a best practice pathway to support improvements in Indigenous Australians' mental health and well-being: a qualitative study. BMJ Open. 2015;5(8).
Williams R, Lawrence C, Wilkes E, Shipp M, Henry B, Eades S, et al. Sexual behaviour, drug use and health service use by young Noongar people in Western Australia: a snapshot. Sexual health. 2015;12(3):188-93.
**Stage 3**
Gowing CJ, McDermott KM, Ward LM, Martin BL. Ten years of trauma in the 'top end' of the Northern Territory, Australia: a retrospective analysis. International emergency nursing. 2015;23(1):17-21.
Johnson DR, McDermott RA, Clifton PM, D'Onise K, Taylor SM, Preece CL, et al. Characteristics of Indigenous adults with poorly controlled diabetes in north Queensland: implications for services. BMC public health. 2015;15:325.
Kendall E, Barnett L. Principles for the development of Aboriginal health interventions: culturally appropriate methods through systemic empathy. Ethnicity & health. 2015;20(5):437-52.
Kickett-Tucker CS, Christensen D, Lawrence D, Zubrick SR, Johnson DJ, Stanley F. Development and validation of the Australian Aboriginal racial identity and self-esteem survey for 8-12 year old children (IRISE_C). International journal for equity in health. 2015;14:103.
Lowell A, Kildea S, Liddle M, Cox B, Paterson B. Supporting aboriginal knowledge and practice in health care: lessons from a qualitative evaluation of the strong women, strong babies, strong culture program. BMC pregnancy and childbirth. 2015;15:19.
O'Grady KA, Dunbar M, Medlin LG, Hall KK, Toombs M, Meiklejohn J, et al. Uptake of influenza vaccination in pregnancy amongst Australian Aboriginal and Torres Strait Islander women: a mixed-methods pilot study. BMC research notes. 2015;8:169.
