# Supplementary material for: Assessing the quality of health research from an Indigenous perspective: the Aboriginal and Torres Strait Islander quality appraisal tool
Source: BMC Med Res Methodol. 2020 Apr 10;20:79. doi: 10.1186/s12874-020-00959-3 (PMC7147059; doi:10.1186/s12874-020-00959-3)
Supplement: Supplementary file 4 — Additional file 4. [file 12874_2020_959_MOESM4_ESM.pdf]

QUESTIONNAIRE FOR STAGE 3 OF THE PILOTING A TOOL TO CRITICALLY APPRAISE LITERATURE THROUGH AN ABORIGINAL AND TORRES STRAIT ISLANDER LENS STUDY

CREATE

Thank you for assessing the feasibility of the CREATE Critical Appraisal Tool and companion Document which has been designed to appraise the quality of research through an Aboriginal and Torres Strait Islander lens. Your participation will be help ensure that CREATE disseminates a Tool that is easy to interpret and efficient to use. Use of the validated Tool will help to ensure that future research with Aboriginal and Torres Strait Islander people aligns with ethical standards, is relevant and benefits Aboriginal and Torres Strait Islander peoples in Australia.

Having appraised the articles provided to you by the CREATE team, using the CREATE Critical Appraisal Tool, the Companion Document and the other critical appraisal tool of your choice, please answer the following questions.

When you have completed the questionnaire, please send the completed critical appraisal assessments of both articles and questionnaire to Stephen Harfield, CREATE Research Fellow by email: stephen.harfield@sahmri.com

Participant ID: _______________

1. How long did it take you to appraise each of the articles using the CREATE Critical Appraisal Tool?

2. How long did it take you to appraise each of the articles using the critical appraisal tool of your choice? Please state the name of the other critical appraisal tool used.

3. Did you experience all the questions on the CREATE Critical Appraisal Tool as easy to interpret and assess the research against?
   If you answered no to question 3, please identify the questions you found it difficult to interpret and assess, and describe the difficulties you experienced.

4. Did you experience any overlap in the quality criteria/questions on the CREATE Critical Appraisal Tool and the critical appraisal tool of your choice?
   If you answered yes to question 4, please describe the overlaps you experienced.

5. What modifications (if any) could be made to improve the feasibility (ease of use and administration) of:
   5.1 Using the CREATE Critical Appraisal Tool and Companion Doucment as a standalone appraisal tool?

   5.2 Using the CREATE Critical Appraisal Tool and Companion Document together with a the critical appraisal tool of your choice that appraises research from a Western research quality perspective?

6. If the CREATE Critical Appraisal Tool was arranged into domains/topics areas, would this assist with the interpretation of findings from your appraisal? How would you then interpret those results based on the findings?
