# Supplementary material for: Assessing the quality of health research from an Indigenous perspective: the Aboriginal and Torres Strait Islander quality appraisal tool
Source: BMC Med Res Methodol. 2020 Apr 10;20:79. doi: 10.1186/s12874-020-00959-3 (PMC7147059; doi:10.1186/s12874-020-00959-3)
Supplement: Supplementary file 5 — Additional file 5. [file 12874_2020_959_MOESM5_ESM.pdf]

Supplementary File 5 – Additional Appraisal Tools used by Participants in Stage 3 of Piloting

CASP Qualitative Research Checklist[1]

Cochrane Risk of Bias Tool[2]

Cochrane Effective Practice and Organisation of Care Risk of Bias Tool[3]

Effective Public Health Practice Project Quality Assessment Tool for Quantitative Studies[4]

Item Bank for Assessing Risk of Bias and Confounding for Observational Studies of Interventions or Exposures[5]

JBI Critical Appraisal Checklist for Analytical Cross Sectional Studies[6]

JBI Critical Appraisal Checklist for Qualitative Research[6]

JBI Critical Appraisal Checklist for Studies Reporting Prevalence Data[6]

McGill Mixed Methods Appraisal Tool version 2011[7]

Quality Assessment of Diagnostic Accuracy Studies Tool[8]

ROBINS-I ("Risk Of Bias In Non-randomised Studies - of Interventions")[9]

Template for Intervention Description and Replication (TIDieR) checklist and guide[10]

References

1. Critical Appraisal Skills Programme. CASP Qualitative Research Checklist. 2017.
2. Cochrane Methods. Assessing Risk of Bias in Included Studies. 2018. http://methods.cochrane.org/bias/assessing-risk-bias-included-studies#The%20Cochrane%20Risk%20of%20Bias%20Tool (accessed 23/03/2018.
3. Cochrane Effective Practice and Organisation of Care. Suggested risk of bias criteria for EPOC reviews. 2017. http://epoc.cochrane.org/resources/epoc-resources-review-authors (accessed 23/03/2018.
4. Effective Public Health Practice Project. Quality Assessment tool for Quantitative Studies. 2009. http://www.ephpp.ca/PDF/Quality%20Assessment%20Tool_2010_2.pdf (accessed 22/12/2017.
5. Viswanathan M, Berkman ND, Dryden DM, Hartling L. Appendix C - Item Bank for Assessing Risk of Bias and Confounding for Observational Studies of Interventions or Exposures. 2013. https://www.ncbi.nlm.nih.gov/books/NBK154460/ (accessed 22/12/2017.
6. Aromataris E, Munn Z, (Editors). Joanna Briggs Institute Reviewers' Manual. In: Aromataris E, Munn Z, editors. Adelaide, Australia: The Joanna Briggs Institute; 2017.
7. Pluye P, Robert E, Cargo M, et al. Mixed Methods Appraisal Tool (MMAT) – Version 2011. Montreal, Canada: Department of Family Medicine, McGill University, 2011.
8. Whiting PF, Rutjes AW, Westwood ME, et al. QUADAS-2: a revised tool for the quality assessment of diagnostic accuracy studies. *Annals of internal medicine* 2011; **155**(8): 529-36.
9. Sterne JA, Hernán MA, Reeves BC, et al. ROBINS-I: a tool for assessing risk of bias in non-randomised studies of interventions. *BMJ* 2016; **355**.
10. Hoffmann TC, Glasziou PP, Boutron I, et al. Better reporting of interventions: template for intervention description and replication (TIDieR) checklist and guide. *BMJ : British Medical Journal* 2014; **348**.
